# Immune Implications of Cholesterol-Containing Lipid Nanoparticles

**DOI:** 10.1021/acsnano.4c06369

**Published:** 2024-10-10

**Authors:** Patricia Ines Back, Minzhi Yu, Shadan Modaresahmadi, Sahelosadat Hajimirzaei, Qisheng Zhang, Md Rakibul Islam, Anna A. Schwendeman, Ninh M. La-Beck

**Affiliations:** † Department of Immunotherapeutics and Biotechnology, Jerry H. Hodge School of Pharmacy, Texas Tech University Health Sciences Center, Abilene, Texas 79601, United States; ‡ Department of Pharmaceutical Sciences, College of Pharmacy, University of Michigan, North Campus Research Complex, 2800 Plymouth Road, Ann Arbor, Michigan 48109, United States; § Division of Chemical Biology and Medicinal Chemistry, Eshelman School of Pharmacy, University of North Carolina at Chapel Hill, Chapel Hill, North Carolina 27599, United States; ∥ Biointerfaces Institute, University of Michigan, North Campus Research Complex, 2800 Plymouth Road, Ann Arbor, Michigan 48109, United States; ⊥ Department of Pharmacy Practice, Jerry H. Hodge School of Pharmacy, Texas Tech University Health Sciences Center, Abilene, Texas 79601, United States

**Keywords:** lipid nanoparticle, liposome, oxysterol, cholesterol, macrophage, immune modulation, lipid metabolism, cholesterol oxidation

## Abstract

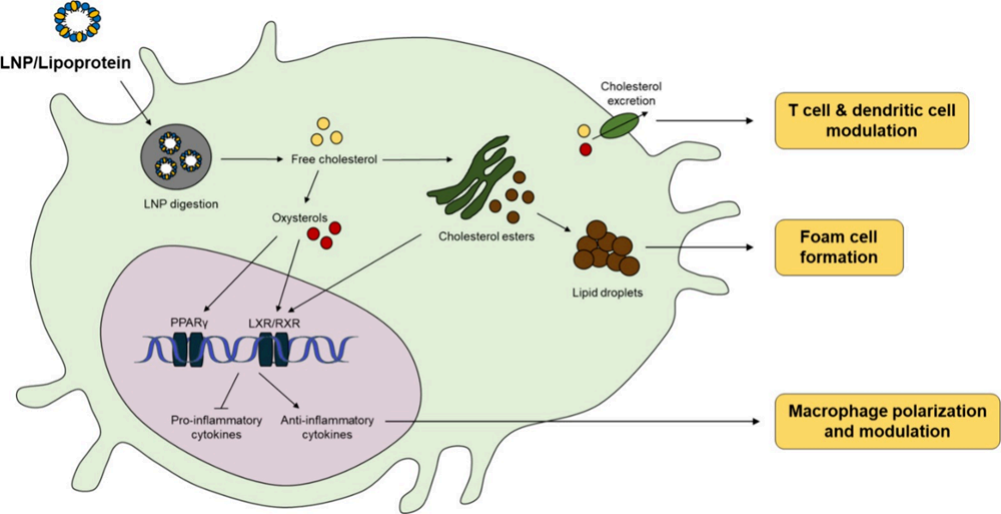

The majority of clinically approved nanoparticle-mediated therapeutics are lipid nanoparticles (LNPs), and most of these LNPs are liposomes containing cholesterol. LNP formulations significantly alter the drug pharmacokinetics (PK) due to the propensity of nanoparticles for uptake by macrophages. In addition to readily engulfing LNPs, the high expression of cholesterol hydroxylases and reactive oxygen species (ROS) in macrophages suggests that they will readily produce oxysterols from LNP-associated cholesterol. Oxysterols are a heterogeneous group of cholesterol oxidation products that have potent immune modulatory effects. Oxysterols are implicated in the pathogenesis of atherosclerosis and certain malignancies; they have also been found in commercial liposome preparations. Yet, the *in vivo* metabolic fate of LNP-associated cholesterol remains unclear. We review herein the mechanisms of cellular uptake, trafficking, metabolism, and immune modulation of endogenous nanometer-sized cholesterol particles (i.e., lipoproteins) that are also relevant for cholesterol-containing nanoparticles. We believe that it would be imperative to better understand the *in vivo* metabolic fate of LNP-associated cholesterol and the immune implications for LNP-therapeutics. We highlight critical knowledge gaps that we believe need to be addressed in order to develop safer and more efficacious lipid nanoparticle delivery systems.

## 1 Pharmacological Properties of Lipid Nanoparticles and the Role of Macrophages

### 1.1 Lipid Nanoparticles (LNPs) as Biocompatible Drug Delivery Systems

Nanoparticles are engineered nanometer-sized particles ranging from 10 to less than 1,000 nm that are commonly used as carriers for therapeutic or diagnostic molecules. Typically, the cargo is within the particle-matrix, adsorbed, or conjugated into its surface.^1^ Numerous organic and inorganic particles have been developed over the years;^1^ nonetheless, lipid nanoparticles have been the most successful carriers due to high biocompatibility and biodegradability of their components.^1^

**Figure 1:**
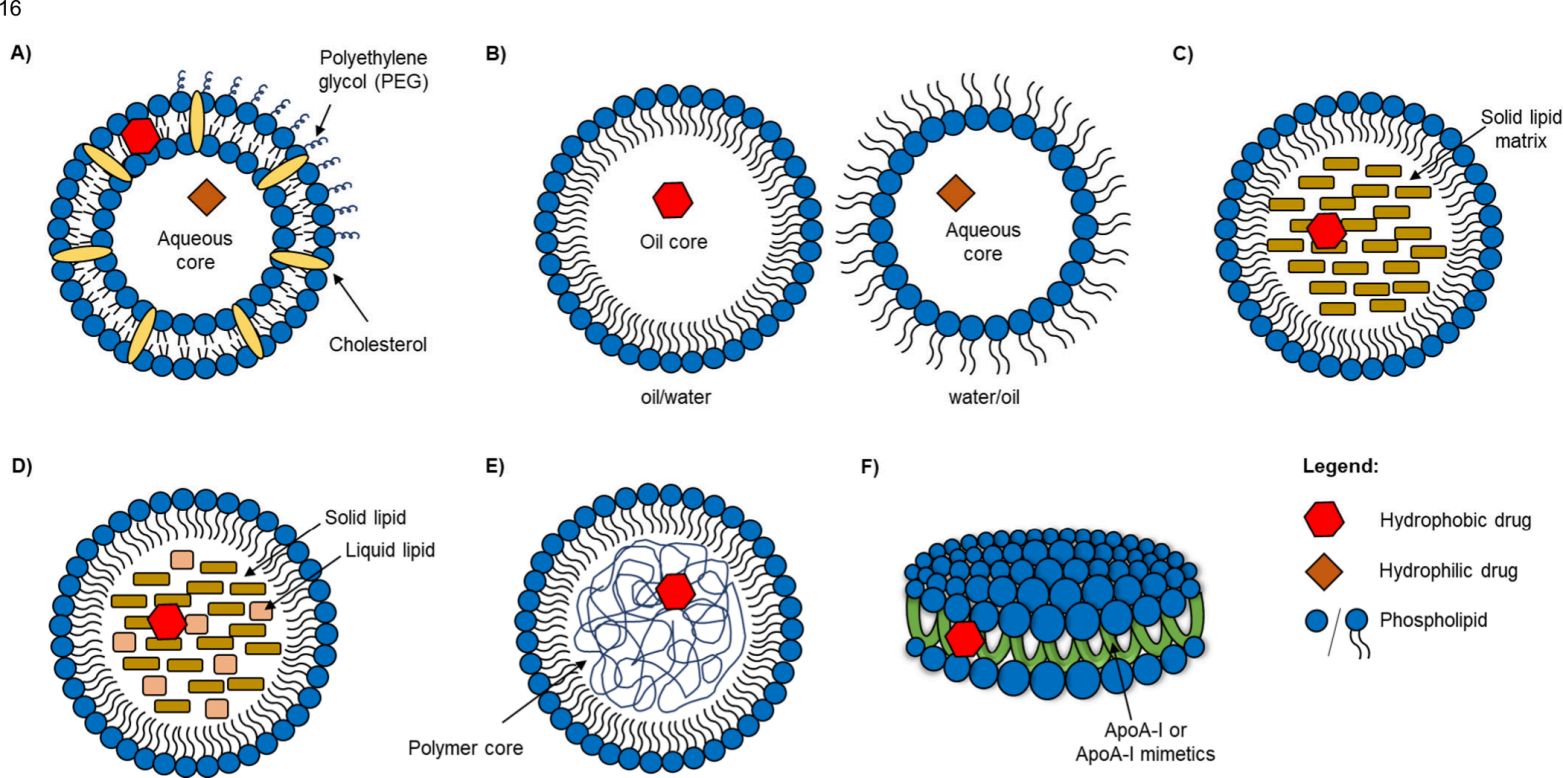
Common structure and components of lipid nanoparticles. A) Liposome, B) nanoemulsions, C) solid lipid nanoparticles, D) nanostructured lipid carriers, E) polymer–lipid hybrid nanoparticles, and F) protein–lipid nanoparticles (sHDL).

Lipid nanoparticles (LNPs) are generally divided into liposomes, lipid nanoemulsions, solid lipid nanoparticles (SLNs), nanostructured lipid carriers (NLCs), polymer–lipid hybrid nanoparticles (PLNs), and protein–lipid nanoparticles (e.g., synthetic high-density lipoproteins, sHDLs). Among the different types of LNPs (Figure 1), liposomes are the most studied nanocarrier due to their biocompatibility, bioavailability, stability, and high drug loading capacity.^2^ They are characterized as spherical vesicles in aqueous media composed of one or more lipid bilayers^2^ usually consisting of phospholipids and cholesterol (Figure 1A).^3^ Since liposomes have both hydrophilic and lipophilic properties, they allow versatile encapsulation and delivery of substances with a wide range of solubility as measured by log P,^4^ with multilamellar liposomes facilitating the incorporation of higher amounts of lipophilic molecules into the vesicle membrane due to increased surface area.^5^

Nanoemulsions are formed by dispersion of oil droplets in an aqueous phase (o/w) or water droplets dispersed in an oil phase (w/o) stabilized by surfactants used for encapsulation of lipophilic or hydrophilic drugs, respectively (Figure 1B).^6,7^ SLNs are comprised of a solid lipid matrix and a surface stabilizer, offering lower mobility of hydrophobic drug molecules within the matrix compared to liquid lipids, used as a strategy on controlled release formulations (Figure 1C).^1^ Alternatively, NLCs contain a combination of liquid and solid lipids in their core, resulting in higher matrix stability and drug loading (Figure 1D),^6^ while PLNs combine a lipid shell with a polymer core (Figure 1E).^6^ Protein–lipid nanoparticles are exemplified by synthetic high-density lipoproteins (sHDLs).^8^ Mimicking the structure of endogenous pre-β HDLs, sHDLs are typically composed of phospholipids and apolipoproteins or their mimetic peptides (Figure 1F).^8^ Unlike liposomes, sHDLs typically present a discoidal structure with a lipid bilayer stabilized by the protein or peptide component.^9^ As drug-free nanoparticles, sHDLs can exert functions similar to endogenous HDLs.^10^ sHDLs can also serve as a drug delivery system, as the lipid bilayer structure allows the encapsulation of lipophilic small molecule drugs.^11^

Together, LNPs offer a series of advantages due to their bioavailability, increased shelf life, controlled release, improved drug absorption, dissolution, and an easy process to scale up production compared to other types of nanoparticle delivery systems.^12^ Thus, in addition to enhancing delivery of hydrophilic and hydrophobic small molecules, nucleic acids, and proteins,^13^ LNPs have been exploited in pharmaceutical formulations to reduce drug degradation,^13^ promote sustained drug release, and increase drug solubility,^2,4^ safety,^14^ and systemic circulation time.^13,15^

Physical and chemical stability of the lipid components are critical for effective LNP drug delivery, particularly for liposomes, as an unstable lipid membrane can result in excessive or premature drug leakage.^4^ They also influence particle size, electrical charge, membrane fluidity, and formulation stability.^2^ Triglycerides are commonly used as lipid matrixes in nanoemulsions to increase the solubilization of lipophilic drugs. However, in multivesicular liposomal formulations, such as Exparel, they reside between bilayer spaces or form oil droplets dispersed in the multivesicular liposomes, acting as space filling and assisting sustained drug delivery.^16^

Phospholipids are the most commonly employed amphophilic lipids formed by a hydrophilic head and hydrophobic acyl chain linked to an alcohol group that confer self-assembly, emulsifying, and wetting characteristics to the particle system.^17^ In water, phospholipids can form micelles, liposomes, or hexagonal phases, thus being used in some LNP formulations to increase the water-solubility of pharmaceutical compounds.^17^ The choice of phospholipid varies according to their cargo, in general aiming to improve drug pharmacokinetics and increase intracellular drug delivery.^18^ Generally, liposomal instability is greater with highly unsaturated phospholipids which are associated with higher membrane permeability,^2^ while the use of saturated phospholipids results in a higher phase transition temperature,^19^ that is associated with more membrane rigidity and lower membrane permeability.^2^ Thus, most commercial liposome formulations commonly use saturated phospholipids to achieve stable liposomes with minimal drug leakage.^20^ However, unsaturated phospholipids and ionizable lipids have better intracellular drug delivery of nucleic acid cargo and are used in applications such as mRNA vaccine delivery. Additionally, the type of phospholipid can also influence the LNP morphology. For instance, DSPC (1,2-distearoyl-*sn*-glycero-3-phosphocholine) leads to the formation of spherical structures, while DPSM (egg sphingomyelin) forms a double layer membrane.^19^ Among phospholipids, 1,2-dioleoyl-*sn*-glycero-3-phosphoethanolamine (DOPE) and saturated phosphatidylcholine (PC) lipids, such as DSPC and l-α-phosphatidylcholine (HSPC), are the most utilized. DOPE is an unsaturated lipid used to improve intracellular delivery of nucleic acids, while DSPC, as a saturated lipid with higher melting temperature (*T*_m_), enables a longer circulation of the carrier in the bloodstream and overall membrane stability.^18^ Indeed, liposomes made with HSPC show tighter membrane packing.^21^ Alternatively, phosphatidylethanolamines (PE) are lipids that can have both saturated and unsaturated tails which are used to stabilize lipid bilayer structures but cannot form bilayers by themselves.^17^

The addition of polyethylene glycol (PEG) to PE (e.g., DSPE–PEG_2000_) is often used in liposome formulations to prolong circulation half-life.^17,22,23^ PEG is used to reduce opsonization and uptake by the reticuloendothelial system (RES), thereby conferring “stealth properties” to the nanoparticles.^22,23^ Nonetheless, its addition to the liposomes requires optimization, as it has the potential to paradoxically increase clearance from the blood through a phenomenon called “accelerated blood clearance” (ABC), which is believed to be mediated by anti-PEG antibodies. Moreover, high concentrations of PE–PEG derivatives may induce the formation of micelles over liposomes.^17^ Overall, the phospholipid headgroup size, phase transition temperature, hydrocarbon saturation, ionic strength, pH, and the incorporation of molecules or divalent cations changes the conformation of the self-assembly carrier that is formed.^17^

Another common component of LNPs, especially liposomes, is cholesterol.^18^ Cholesterol is a major component of cell membranes and is responsible for their stability, fluidity, and permeability.^24,25^ As such, cholesterol is also used in liposomes for similar stabilizing functions, with a controllable and reproducible release for a variety of drugs achieved by a 70:30 phospholipid:cholesterol ratio.^4^ When present in the lipid bilayer, cholesterol increases its organization, impacting liposome shape, size, and drug release.^4^ It can also modify the fluidity of the lipid bilayer and resistance to shear stress,^26^ depending on the starting properties of the lipid matrix.^27^ Cholesterol modifies phospholipid molecule packing^28^ and aggregation resistance^29^ and reduces the lipid bilayer permeability to electrolytes and non-electrolyte solutes,^28,30^ thus making the formulation more stable. Overall, considering the similarities between cellular structures and LNPs, these nanocarriers achieve biocompatibility by mimicking the physicochemical, mechanical, and biological properties of cell membranes for drug delivery.^31^

### 1.2 Clinically Relevant LNPs

Many promising drug discoveries in the preclinical phase fail to enter clinical trials due to poor biopharmaceutical and pharmacokinetic properties, such as low aqueous solubility/stability and rapid metabolism and renal clearance.^32^ In this sense, nanoparticles have been developed as drug carriers to ameliorate these pharmaceutical limitations.^32^ For example, DepoDur encapsulates morphine sulfate in a multivesicular lipid-based liposome which allows sustained-release of the drug into the epidural space.^33^ Visudyne contains verteporfin, a lipophilic compound, stabilized in a unilamellar liposome that is used as a photosensitizer for photodynamic therapy.^33^ Mepact is composed of mifamurtide or 1,2-dipalmitoyl-glycero-3-muramyl tripeptide-phosphatidylethanolamine, also a lipophilic drug, covalently linked to liposomal PE (lipid–drug conjugation).^34^

In addition to the delivery of small molecule drugs, nanoparticles can be exploited for the delivery of nucleic acids. In particular, naked RNA is highly unstable due to the presence of nucleases in the serum and a rapid renal clearance, resulting in an elimination half-life within minutes.^35^ Aiming to increase stability, LNPs created to deliver oligonucleotides are solid nanoparticles that, in addition to cholesterol and a PEG–lipid, contain a cationic lipid that neutralizes the anionic charged RNA.^5^ Such modification allows efficient RNA encapsulation and release from endosomal uptake, improving circulation time, tissue distribution, and intracellular drug delivery of RNA drugs such as patisiran (Onpattro).^5^

Currently, the majority of clinically approved LNPs are liposomes, with more than 20 liposomal products available worldwide, of which nearly half are approved for the treatment of solid cancers and hematologic malignancies (Table 1). One major reason for this is that liposomal encapsulation of chemotherapeutics significantly improves tolerability by decreasing drug extravasation at off-target tissue sites. Pegylated liposomal doxorubicin (Doxil) is the most widely used liposomal formulation in cancer and was shown to mitigate the dose-limiting cardiotoxicity associated with conventional unencapsulated doxorubicin.^14^ Another successful case outside the context of cancer is amphotericin B, a broad-spectrum antifungal agent that is associated with significant nephrotoxicity, infusion-related reactions, and poor water-solubility, limiting its use in the clinic. The incorporation of amphotericin B into a liposome bilayer (AmBisome) significantly reduces its toxicity.^36^ Indeed, AmBisome significantly decreases invasive fungal infections, nephrotoxicity, and treatment delay/discontinuation compared to non-liposomal amphotericin B.^37^ Amphotec presents the same benefits by stabilizing amphotericin B in disc-like lipid particles, reducing acute toxicity, hemolysis, and lipoprotein binding.^33^

**Table 1:** Clinically Approved LNPs and Therapeutic Areas (Conditions/Diseases)^a^

**Clinically approved liposomes (approval year)**	**Active agent**	**Lipid composition**	**LNP carrier**	**Indication**
Diprivan (1989)^41,42^	Propofol	Soybean oil (100 mg/mL):glycerol (22.5 mg/mL):egg lecithin (12 mg/mL)	Nanoemulsion	Sedation or anesthesia
Epaxal (1993)^33,41,43^	Inactivated hepatitis A virus (strain RGSB)	DOPC:DOPE (75:25 molar ratio)	Liposome	Hepatitis A
Doxil/Caelyx (1995)^33,43,44^	Doxorubicin	HSPC:cholesterol:PEG2000-DSPE (56:39:5 molar ratio)	Liposome	Ovarian and breast cancer, AIDS-related Kaposi’s sarcoma, and multiple myeloma (in combination with bortezomib)
Abelcet (1995)^33,43^	Amphotericin B	DMPC:DMPG (7:3 molar ratio)	Liposome	Invasive severe fungal infections
DaunoXome (1996) discontinued^33,43^	Daunorubicin	DSPC:cholesterol (2:1 molar ratio)	Liposome	AIDS-related Kaposi’s sarcoma
Amphotec (1996) discontinued^33,43^	Amphotericin B	Cholesteryl sulfate:amphotericin B (1:1 molar ratio)	Liposome	Severe fungal infections
AmBisome (1997)^33,43^	Amphotericin B	HSPC:DSPG:cholesterol:amphotericin B (2:0.8:1:0.4 molar ratio)	Liposome	Presumed fungal infections
Fungizone (1997) discontinued^41,45^	Amphotericin B	DMPC:DMPG:amphotericin B (7:3:5 molar ratio)	Liposome	Systemic fungal infections
Inflexal V (1997) discontinued^33,41,43^	Inactivated hemagglutinin of influenza virus strains A and B	DOPC:DOPE (75:25 molar ratio)	Liposome	Influenza
Depocyt (1999) discontinued^33,46,47^	Cytarabine	Cholesterol, triolein, DOPC, and DPPG (11:1:7:1 molar ratio)	Liposome	Lymphomatous meningitis
Rapamune (oral solution) (1999) discontinued^41,48^	Rapamycin (sirolimus)	PC, propylene glycol, glycerides, ethanol, soy fatty acids, ascorbyl palmitate, and polysorbate 80	Liposome	Immunosuppressant
Myocet (2000)^33,43^	Doxorubicin	EPC:cholesterol (55:45 molar ratio)	Liposome	Metastatic breast cancer in combination with cyclophosphamide
Visudyne (2000)^33,43,46^	Verteporphin	EPG:DMPC (3:5 molar ratio)	Liposome	Choroidal neovascularization due to age-related macular degeneration, pathologic myopia, or presumed ocular histoplasmosis
DepoDur (2004) discontinued^33,43,46^	Morphine sulfate	Cholesterol, triolein, DOPC, and DPPG (11:1:7:1 molar ratio)	Liposome	Pain management
Mepact (2004 EMA approval only)^33^	Mifamurtide	DOPS:POPC (3:7 molar ratio)	Liposome	High-grade, resectable, non-metastatic osteosarcoma
Exparel (2011)^33,43,49^	Bupivacaine	DEPC (8.2 mg/mL), DPPG (0.9 mg/mL), cholesterol (4.7 mg/mL), and tricaprylin (2.0 mg/mL)	Liposome	Pain management
Marqibo (2012) discontinued^33^	Vincristine	SM:cholesterol (60:40 molar ratio)	Liposome	Acute lymphoblastic leukemia (FDA approval withdrawn in 2021)
Lipodox (2013) discontinued^43,50^	Doxorubicin	DSPC:cholesterol:PEG_2000_-DSPE (56:39:5 molar ratio)	Liposome	Breast and ovarian cancer
Lipusu (2003 approval in China)^43^	Paclitaxel	PC:cholesterol	Liposome	Ovarian, breast, and non-small-cell lung cancer
Ikervis (2015)^41,51^	Cyclosporine A	Medium-chain triglycerides, tyloxapol, and poloxamer 188	Nanoemulsion	Keratitis in patients with dry eye disease
Mosquirix vaccine (initial approved 2015; now available in Cameroon, Malawi, Kenya, Sierra Leone, Ghana, and Liberia)^43,52−54^	*P. falciparum* circumsporozoite protein fused with hepatitis B surface antigen (RTS) and combined with hepatitis B surface antigen (S)	DOPC:cholesterol	Liposome	Malaria caused by *Plasmodium falciparum*
Onivyde (2015)^33^	Irinotecan	DSPC:MPEG_2000_:DSPE (3:2:0.015 molar ratio)	Liposome	Metastatic adenocarcinoma of the pancreas in combination with fluorouracil and leucovorin
Vyxeos (2017)^43,55^	Daunorubicin and cytarabine	DSPC:DSPG:cholesterol (7:2:1)	Liposome	Acute myeloid leukemia
Arikayce (2018)^56^	Amikacin	DPPC:cholesterol	Liposome	*Mycobacterium avium* complex lung disease
Onpattro (2018)^43,57^	Patisiran, a transthyretin-directed small interfering RNA	Cholesterol:DSPC:Dlin-MC3-DMA:PEG_2000_-C-DMG	Lipid nanoparticle	Polyneuropathy caused by hATTR amyloidosis
Spikevax (2022)^58^	Messenger ribonucleic acid (mRNA)	SM-102, PEG_2000_ DMG, cholesterol, and DSPC	Lipid nanoparticle	Immunization for coronavirus disease 2019 (COVID-19)
Comirnaty (2021)^59^	Messenger ribonucleic acid (mRNA) against SARS-CoV-2	0.43 mg ALC-0315, 0.05 mg mPEG-DTA, 0.09 mg DSPC, and 0.2 mg cholesterol	Lipid nanoparticle	Immunization for coronavirus disease 2019 (COVID-19)

a HSPC (hydrogenated soy phosphatidylcholine); PEG (polyethylene glycol); DSPE (distearoyl-*sn*-glycero-phosphoethanolamine); DSPC (distearoylphosphatidylcholine); DOPC (dioleoylphosphatidylcholine); DPPG (dipalmitoylphosphatidylglycerol); EPC (egg phosphatidylcholine); EPG (egg phosphatidylglycerol); DOPS (dioleoylphosphatidylserine); POPC (palmitoyloleoylphosphatidylcholine); SM (sphingomyelin); MPEG (methoxy polyethylene glycol); DMPC (dimyristoylphosphatidylcholine); DMPG (dimyristoylphosphatidylglycerol); DSPG (distearoylphosphatidylglycerol); DEPC (dierucoylphosphatidylcholine); DOPE (ioleoyl-*sn*-glycero-phophoethanolamine); DMG (dimyristoyl glycerol); ALC-0315 (4-hydroxybutyl)azanediyl)bis(hexane-6,1-diyl)bis(2-hexyldecanoate); mPEG-DTA ([(polyethylene glycol)-2000]-*N*,*N*-ditetradecylacetamide); PC (phosphatidylcholine).

No synthetic lipoprotein has yet been approved, but there are two currently in late phase clinical trials: CSL 112 and CER-001. CSL112, composed of plasma purified apolipoprotein (Apo) A-I (ApoA-I) and phosphatidylcholine, has just finished a phase 3 trial for the reduction of major adverse cardiovascular events in the 90-day high-risk period post-acute myocardial infarction.^38^ CER-001 is comprised of recombinant ApoA-I, egg sphingomyelin, and DPPG (dipalmitoylphosphatidylglycerol) and is currently in phase 2 trials for sepsis with high risk of developing acute kidney injury, lecithin cholesterol transferase (LCAT) deficiency (kidney dysfunction), and ophthalmologic disease.^39,40^

### 1.3 Macrophages and LNP Pharmacokinetics

Formulating a drug in LNPs significantly alters the drug pharmacokinetics (PK). The PK of an LNP carrier dictates the overall PK profile until the drug is released from the LNP carrier. Typically, LNP drug delivery is associated with increased drug accumulation in the spleen and liver, due to the propensity of nanoparticles for uptake by macrophages in the reticuloendothelial system (RES), also known as the mononuclear phagocyte system (MPS).^33,60^ Macrophages are innate immune cells responsible for engulfing bacteria, cellular debris, and foreign particles within minutes after exposure, thereby enabling infection containment and clearance.^13^ They are present in most body tissues and can originate from bone marrow derived monocytes, from yolk-sac cells during embryogenesis, or from fetal liver monocytes.^61^ Tissue-resident macrophages (e.g., Kupffer cells, alveolar macrophages, spleen red pulp macrophages, brain-resident microglia, epidermal Langerhans cells) have a longer life span and self-renewal properties compared to their circulating precursors (monocytes).^62^ Macrophages are highly phagocytic; they initiate inflammatory responses as well as tissue development, homeostasis, and repair,^61^ aiming to defend the host and maintain tissue integrity.^62^ These cells show high plasticity and heterogeneity and, depending on the local tissue environment, express different phenotypes ranging from the classically activated M1 phenotype, associated with pro-inflammatory functionality, to the alternatively activated M2 phenotype associated with pro-healing/anti-inflammatory states inducing local angiogenesis and tissue neogenesis.^13,62^

Once administered, LNPs are quickly covered with proteins from body fluid, forming a protein corona. The composition of the protein corona is dependent on many patient-specific factors (e.g., comorbidities, sex, age) as well as on the physicochemical characteristics of the LNP system. The most common constituents include albumin, immunoglobulins, complement proteins, and apolipoproteins such as ApoA-I, ApoB-100, and ApoE.^63^ The protein corona alters the surface properties of LNPs, and it affects their interactions with macrophages and other cells. Some protein corona components, such as albumin, show inhibitory effects on cellular uptake of LNPs.^64^ In contrast, complement proteins adsorbed to the surface of nanoparticles can be recognized by complement receptors on macrophages, promoting the engulfment of LNPs.^13,65^ The apolipoproteins enriched in the protein corona also regulate the interactions between LNPs and macrophages. For example, it has been reported that surface adsorbed ApoA-I significantly reduced the macrophage uptake of lipid nanoparticles.^66^ Enrichment of ApoB and ApoE, on the other hand, enhanced the macrophage uptake through recognition by lipoprotein receptors such as low-density lipoprotein receptors (LDLRs) and scavenger receptors.^67,68^ Interestingly, it has been observed that the protein corona greatly enhanced the nanoparticle uptake by M2 macrophages,^69^ which may be attributed to the different lipid receptor expression profiles between M1 and M2 macrophages. Such an interplay between LNPs, protein corona, and macrophages affects the elimination and biodistribution of LNPs, thus directly impacting the pharmacokinetics and pharmacodynamics of LNPs.

Macrophages recognize proteins, including complement activating proteins (opsonins), that adsorb to the surface of nanoparticles, promoting their engulfment and subsequent clearance from the circulation.^13,65^ Prolonged plasma drug circulation and concentrations can be accomplished by drug encapsulation in stable nonleaky LNPs with small size and “stealth” modifications such as pegylation, which can decrease or slow uptake by the MPS.^33^ Females typically have lower MPS function than males; thus, after intravenous administration, they show slower liposomal clearance rates than males.^65^ The interactions between LNPs and the MPS often limit the accumulation of nanoparticles at target tissue sites, while increasing accumulation in macrophages which can lead to inflammatory and toxic responses.^13^ The activation of tissue resident macrophages can induce surface receptor rearrangements that increase nanoparticle recognition and internalization.^13^ Since LNPs increase delivery of drugs to the MPS, this can also enhance the drug-associated immunomodulatory effects on macrophages.^32,70,71^

Nanoparticle uptake and biological responses vary depending on the macrophage differentiation and polarization states, impacting formulation biodistribution, delivery, tissue accumulation, and clearance.^72^*In vitro*, liposome uptake by macrophages happens within 2 h, with the development of a proinflammatory M1-like profile even in previously M2-polarized macrophages.^73^ There is also an increase in acyl-CoA:diacylglycerol acyltransferase (DGAT-1 and DGAT-2) expression in M0 and M2 macrophages that correlates with increased accumulation of lipid droplets, suggesting higher lipid droplet biogenesis in these cell types.^73^*In vivo*, systemically administered liposomes accumulate not only in the liver and spleen but also in bone marrow macrophages.^73^ Following the administration of 200 mg/kg of liposomes, these LNPs promote myeloid differentiation in the bone marrow and development of a “foam” cell phenotype reminiscent of the inflammatory macrophages that are observed in atherosclerotic plaques.^73^ Indeed, due to abnormal lipid droplet biogenesis at the endoplasmic reticulum (ER), liposomes induce ER stress and upregulate inflammatory genes through the nuclear factor-kappa B (NF-κB) signaling pathway, leading to a significant increase of inflammatory cytokine (IL-6 and IL-1β) secretion.^73^ Interestingly, liposomes that do not contain cholesterol also do not stimulate the formation of “foam” cells.^74^ Together, these findings support a bidirectional interaction between LNPs and macrophage functionality and suggest that the underlying molecular mechanisms are linked to the cholesterol component of the LNP.

### 1.4 Cholesterol Homeostasis Intersects with Immune Regulation in Macrophages

**Figure 2:**
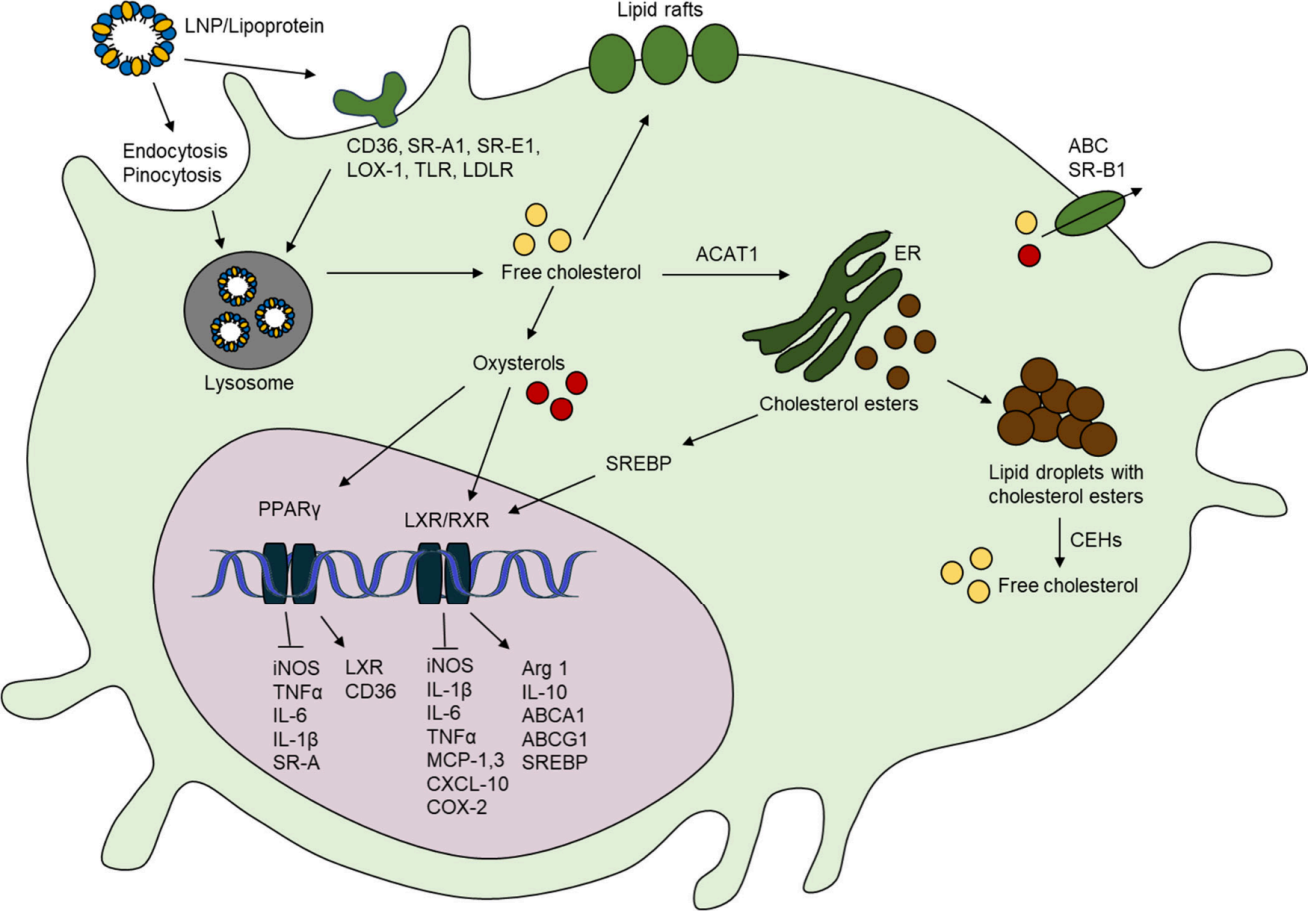
Summary of LNP and lipoprotein trafficking in macrophages showing uptake, cholesterol homeostasis, and immune regulation. ABC, ATP-binding cassette transporter; ACAT1, acetyl-coenzyme A acetyltransferase-1; ApoER, apolipoprotein E receptor; Arg 1, arginase 1; CEHs, cholesterol ester hydrolases; COX, cyclo-oxygenase; CXCL, chemokine (C-X-C motif) ligand; ER, endoplasmic reticulum; IL, interleukin; iNOS, inducible nitric oxide synthase; LDLR, low-density lipoprotein receptor; LNP, lipid nanoparticle; LOX-1, lectin-like oxidized low-density lipoprotein receptor-1; LXR, liver X receptor; MCP, monocyte chemoattractant protein; PPAR, peroxisome proliferator-activated receptor; RXR, retinoid X receptor; SR, scavenger receptor; TLR, toll-like receptor; TNF, tumor necrosis factor; VLDLR, very low-density lipoprotein receptor.

Once ingested, cholesterol is packed into chylomicrons in intestinal epithelial cells, which are then transported into the lymphatic system where they acquire apolipoprotein (Apo) B before entering the circulation.^61^ Cholesterol is then transported through the body by these lipoproteins, which are nanometer-sized lipid particles that interact with macrophages through similar mechanisms as LNPs. Most of the cholesterol is transported from peripheral tissues to the liver by high-density lipoproteins (HDLs) and from the liver to the peripheral tissues by low-density lipoproteins (LDLs).^75^ Macrophages are the primary cells responsible for taking up lipoproteins from circulation and from dying cells in order to eliminate excess cholesterol from the body.^61^ Through phagocytosis, micropinocytosis, and receptor-mediated pathways, macrophages take up lipoproteins, digest the lipids in the lysosome, and produce free fatty acids and free cholesterol (Figure 2).^61^ While CD36 is involved in lipoprotein uptake, blockade of this receptor does not impact lipid uptake by macrophages,^76^ suggesting additional uptake mechanisms. Oxidation of the cholesterol in LDL (oxidized LDL) results in interactions with macrophages through scavenger receptors on the cell surface, as well as through pathogen-associated molecular pattern-recognition receptors that include the Toll-like receptors TLR4, TLR3, and TLR6.^74^ TLRs have been linked to alterations of the metabolic state of macrophages, including decreased cholesterol efflux and immune response through the activation of NF-kB.^74^ Activation of NF-kB signaling and IFN-α in the presence of TLR ligands (e.g., exogenous lipids such as LNPs) leads to PPARα activation and foam cell development.^74^

Inflammatory M1 macrophages can quickly switch their metabolic phase from glycolysis to production of amino acids for protein synthesis, ribose for nucleotides production, fatty acids for inflammatory mediator production, and NADPH for reactive oxygen species (ROS) and nitric oxide (NO).^62,77,78^ Pyruvate, a glycolysis product, is used in the tricarboxylic acid (TCA or Krebs) cycle, leading to oxidative phosphorylation and ATP production, which are used for ROS synthesis necessary for inflammation.^62^ Citrate produced during TCA is converted into acetyl-CoA, leading to fatty acid synthesis (FAS) and lipogenesis and being used for changes in the plasma membrane to favor inflammatory signaling and production of inflammatory mediators (e.g., NO). Succinate, a product from the TCA cycle, is pro-inflammatory and can increase stabilization of LPS-induced hypoxia inducible factor 1 alpha (HIF1α). HIF1α interacts with IL-1β and IL-6, promoting inflammasome activation and/or activation of STAT3, while also supporting the production of mitochondrial ROS due to increased mitochondrial oxidation through succinate dehydrogenase.^62,76^

In contrast, M2 macrophages require higher mitochondrial oxidative respiration (OXPHOS), which is driven by fatty acid oxidation (FAO) and glucose.^62,79^ These processes take longer to initiate but generate more ATP than glycolysis (∼34 ATP molecules per glucose versus 2 ATP), thus supporting long-term survival of macrophages and cellular functions required for antiparasite responses, tissue/wound healing, and homeostasis.^62^ M2 macrophages are primarily induced by IL-4 but can also be activated by IL-10, IL-13, glucocorticoids, and M-CSF, resulting in different metabolic patterns.^62^ For instance, in the intestine, macrophages are constantly exposed to IL-10 produced by regulatory T cells (Tregs), leading to the suppression of glucose uptake by interrupting the glucose transporter GLUT1 translocation to the cell surface and down-regulating enzymes involved in glycolysis.^62^ With changes in the microenvironment stimulus, M2 macrophages can easily switch to M1-like metabolic state.^62^ M1 macrophages have been shown to have higher accumulation of endogenous lipids in the cytoplasm compared to M0 and M2 phenotypes.^73^ However, the administration of liposomes is associated with higher lipid retention in M0 and M2 macrophages.^73^

After internalization, LNPs are disrupted by lysosomal phospholipases, generating free fatty acids and cholesterol.^73^ These then accumulate as lipid droplets that are mainly comprised of triacylglycerols and cholesterol esters,^73,74,80^ which may result in a dysfunctional macrophage phenotype similar to foam cells.^73,74^ Indeed, a direct correlation was observed between the cholesterol amount in liposomes and the severity of foam cell formation, and similar findings have been demonstrated with 80–100 nm exosomes (extracellular lipid bilayer vesicles generated by cells).^74^ Foam cells are typically associated with accumulation of oxidized low-density lipoprotein (oxLDL) but not unoxidized LDL.^74^ Therefore, considering that LDL is an endogenous nanometer-sized lipid particle, it is possible that liposomes similarly undergo metabolism through oxidative pathways. However, there is a lack of studies that track the intracellular metabolic fate of liposomal lipids.

Interestingly, liposomes have been used in the lipid biology field as model systems for cellular membranes to study membrane lipid trafficking, peroxidation, metabolism, and other phenomena,^81,82^ suggesting that liposomal lipids are not inert. Aside from the LNP carrier, the drug cargo can also impact macrophage polarization state, influencing local inflammation and angiogenesis.^70,83^ Yet, how the immune modulatory effects of the carrier alter those of the drug cargo, and *vice versa*, has not been systematically studied. Given that macrophages express high levels of receptors capable of binding LNPs for internalization and high levels of cholesterol hydroxylases, it would be imperative to understand the common mechanisms that regulate cellular LNP interactions, cellular cholesterol handling and metabolism, and immune modulatory effects of oxidized cholesterol (oxysterols).

## 2 Handling of Cholesterol-Containing Lipid Particles in Macrophages

### 2.1 Uptake

The endogenous cholesterol uptake in macrophages is mediated by various receptors (Figure 2).^84^ LDLR is responsible for the endocytosis of unmodified LDLs. Modified LDLs, such as oxidized LDL (oxLDL) and acetylated LDL (acLDL), are recognized and endocytosed by scavenger receptors (SRs), among which the scavenger receptors type A1 (SR-A1) and cluster of differentiation 36 (CD36) account for 75–90% of the uptake of modified LDL.^85^ Located in the lipid raft of cell membranes, SR-A1 recognizes and binds a wide range of modified LDL through its collagen-like domain, resulting in the endocytosis of modified LDLs.^86^ CD36 is a type B scavenger receptor that recognizes a variety of ligands, including oxidized lipids in oxLDL, leading to the endocytosis of oxLDL.^87^ Other scavenger receptors, such as SR-E1 (also known as lectin-type oxidized LDL receptor 1, LOX1), also mediate the uptake of modified LDLs.^88^ The expression of scavenger receptors can be upregulated by factors such as inflammation, which leads to excessive cellular uptake of modified LDLs.^89,90^ The accumulation of excessive LDL-derived lipids and cholesterol can then cause the formation of foam cells, which are the major driving force of atherosclerosis.^89^ Cholesterol-containing liposomes are also internalized by LDLR, CD36, and SR-A1 receptors, suggesting that they may undergo similar intracellular processing as endogenous cholesterol particles such as LDL.^91^

ApoE is one of the main components of very low-density lipoproteins (VLDLs) and high-density lipoproteins (HDLs), and it can also be a component of the protein corona that forms on LNPs. Macrophages express both VLDL receptors (VLDLRs) and ApoE receptors (e.g., ApoER2), with the latter being predominantly expressed in M2-macrophages. Thus, it is likely that cholesterol-containing liposomes may adsorb ApoE and undergo internalization through the ApoE receptors. The uptake of lipid particles and the associated switch from M1 to M2 macrophage phenotype were shown to involve VLDLR and ApoER2.^92^ Onpattro, an LNP used to treat polyneuropathy caused by hereditary transthyretin-mediated amyloidosis (hATTR amyloidosis), has been shown to deliver its siRNA cargo to the liver through the nonspecific adsorption of ApoE onto the LNP and subsequent binding to lipoprotein receptors in hepatocytes.^93^ In addition to the similarities between LNPs and lipoprotein uptake pathways, there are also differences such as a prominent role for clathrin-mediated endocytosis of cholesterol-containing liposomes, whereas caveolin-mediated endocytosis is more common for endogenous lipid particles.^91^ These differences indicate that there are also intracellular trafficking mechanisms that are specific to exogenous LNPs.

### 2.2 Trafficking and Storage

Cholesterol is mainly transported in the esterized form by different lipoproteins in the circulation. Most cholesterol ester is synthesized by lecithin:cholesterol acyltransferase (LCAT).^94^ LCAT preferably interacts with nascent HDLs, converting free cholesterol to cholesterol ester through the transfer of a fatty acid from lecithin to cholesterol.^94^ During circulation, the HDL-associated cholesterol ester may be transferred to LDL or VLDL through cholesteryl ester transfer protein (CETP) in exchange for triglyceride.^95^ Thus, the esterification activity of LCAT plays an important role in maintaining the concentration gradient of cholesterol between cell membranes and cholesterol receptors in the plasma, promoting the efflux of cholesterol from peripheral tissues.

Following internalization (Figure 2), LDL enters the endosomal–lysosomal system where the cholesterol ester associated with LDL is hydrolyzed to free cholesterol by lysosomal acid lipases (LALs).^96^ The free cholesterol is then transported to other organelles, such as the ER, and cell membranes.^97^ After cellular uptake, liposomes similarly localize to endosomal and lysosomal compartments and then are trafficked to the endoplasmic reticulum (ER) and to the Golgi apparatus.^91^ However, there are differences in the trafficking of the liposome phospholipid versus the cholesterol components, with the latter preferentially localizing to the ER.^91^ Liposomes have also been reported to traffic intact to the ER, where fusion with ER membranes can occur.^98^ The post-lysosomal trafficking of free cholesterol is mediated by Niemann–Pick type C1 (NPC1) and NPC2, where the free cholesterol is transported through membrane vesicles or carrier proteins.^99,100^ To prevent the cytotoxicity of free cholesterol, excess free cholesterol in the ER is esterified by acyl coenzyme A:cholesterol acyltransferase-1 (ACAT1) to form non-cytotoxic cholesterol ester.^101^ The cholesterol ester is stored in lipid droplets, a cellular organelle consisting of a neutral lipid core surrounded by phospholipids, free cholesterol, and associated proteins.^102^ Liposomes have also been found to be incorporated into ER-assembled entities such as lipoproteins and lipid droplets.^98^ When the internal cholesterol concentration is low, the transcription factors SREBP2 and SREBP1c are cleaved by SREBP cleavage-activating protein, allowing their translocation from the Golgi apparatus to the nucleus, where they bind to their promoter gene in liver X receptor (LXR), leading to the activation of lipogenic genes.^103^ When the intracellular cholesterol levels are high, the cholesterol esters stored in lipid droplets are hydrolyzed by cholesterol ester hydrolases (CEHs), generating free cholesterol that is subsequently transported out by efflux proteins.^104^

**Figure 3:**
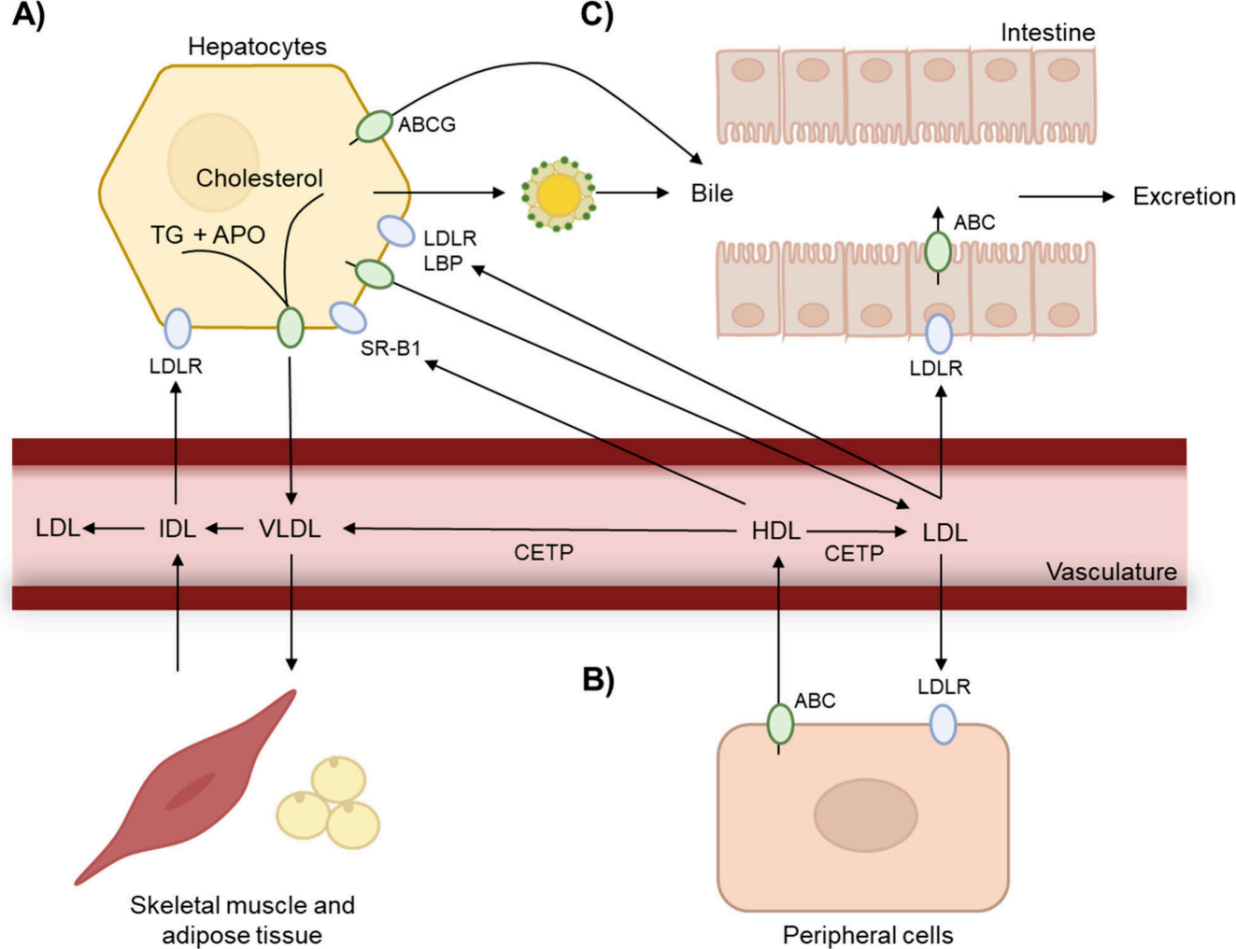
Cholesterol transport and elimination. A) Cholesterol from exogenous (e.g., LNPs and food) or endogenous sources (e.g., liver and peripheral tissues) is combined with apolipoproteins (APOs) and triglycerides (TGs) to form VLDL particles that are secreted into the circulation. Through VLDLR, VLDL enters the adipose tissue, skeletal muscle, heart, and kidneys. VLDL can be converted to intermediate-density lipoproteins (IDLs) by lipoprotein lipase. IDL can be directly taken up by the liver or converted to LDL through the action of hepatic lipases. B) The reverse cholesterol transport (RCT) pathway is where cholesterol is transported in high-density lipoproteins (HDLs) from extrahepatic tissues to the liver. HDL-associated cholesterol ester may be transferred to LDL or VLDL through the cholesteryl ester transfer protein (CETP). C) Cholesterol is excreted through the hepatobiliary pathway as bile or through the transintestinal cholesterol efflux (TICE) pathway.

Reverse cholesterol transport (RCT) (Figure 3), a process where excess cholesterol is removed from peripheral cells and transported to the liver and intestine for elimination, is essential for maintaining cholesterol homeostasis.^105^ In macrophages, the cholesterol efflux is mediated mainly by ATP-binding cassette (ABC) transporters including ABCA1 and ABCG1, but other transporters, such as scavenger receptor class B type I (SR-BI), are also involved.^106^ ABCA1 is located both on cell membranes and on cellular organelles such as endocytic vesicles. It mediates the transport and efflux of lipid-poor apolipoproteins, promoting the assembly of HDL particles.^107^ ABCG1, located in endocytic vesicles, mediates cholesterol trafficking from the ER to the cell membrane with subsequent cholesterol efflux.^108^ Unlike ABCA1, ABCG1 promotes cholesterol efflux to HDL particles, but not lipid-poor lipoproteins.^108^ Liposomes have been found to undergo efflux via ABCA1 and ABCB1, but not ABCG1.^91^ The expression of ABCA1 and ABCG1 is regulated by numerous cholesterol-dependent signaling pathways, such as the liver X receptor (LXR)/retinoid X receptor (RXR) pathway.^109,110^ Activating inflammation-related pathways, such as the JAK2/STAT3 pathway, has been found to compromise the expression and function of ABCA1 and ABCG1, suggesting underlying crosstalk mechanisms between inflammation and cholesterol homeostasis.^109^ Additionally, SR-B1 also contributes to cholesterol efflux from macrophages by facilitating the passive diffusion of cholesterol from the cell membrane to HDL particles. It was proposed that, at low HDL concentrations, SR-B1 could bind to HDL and form a hydrophobic tunnel to facilitate the transfer of cholesterol.^111^ SR-B1 may also redistribute cholesterol on cellular membranes, creating a more favorable environment for cholesterol diffusion.^111^

### 2.3 Excretion

Increasing the cholesterol content of liposomes increases liposome elimination rates.^112^ However, the precise mechanism of excretion is unclear as most studies monitor the released drug cargo rather than track the liposomal cholesterol and lipids directly.^112^ It is probable that the excretion pathways of cholesterol containing liposomes overlap with those for lipoproteins since both are nanometer-sized lipid particles with overlapping cellular trafficking mechanisms as described above and shown in Figures 2 and 3.^112^

The most studied cholesterol excretion pathway is the hepatobiliary pathway, where cholesterol is converted to bile acids and secreted into bile.^113^ In this case, both LDL- and HDL-associated cholesterol (LDL-C and HDL-C) can be internalized by hepatocytes; however, there is a higher contribution of HDL-C to biliary cholesterol excretion.^114^ SR-B1 is the major receptor mediating the uptake of HDL-C, while LDLs can be internalized through various LDL receptors, such as LDLR and LBP (LPS-binding protein).^115,116^ The internalized cholesterol is either converted to bile acid or effluxed by ABCG5 and ABCG8 to the bile.^117^

The transintestinal cholesterol excretion (TICE) pathway, where cholesterol is secreted into the intestinal lumen by the intestinal epithelium, has gained increasing research interest in the past decade.^118^ A recent study estimated that TICE accounts for 35% of the excretion of cholesterol in humans.^119^ In the process of TICE, cholesterol is first taken up by enterocytes at the basolateral side, followed by intracellular trafficking and excretion into the intestinal lumen at the apical side. The exact mechanisms involved in TICE have not been fully elucidated.^120^ ABCA-1 or SR-B1 knockout mice lacking HDL presented TICE similar to that of wildtype counterparts, implying a relatively minor role of HDLs.^121^ Acute inhibition of LDLR by PCSK9 (proprotein convertase responsible for LDL receptor degradation) decreased TICE, suggesting that LDLR and ApoB-containing liposomes might play a role in basolateral cholesterol uptake by enterocytes.^122^ Intriguingly, LDLR knockout mice showed increased TICE, suggesting alternative cholesterol uptake pathways that are yet to be elucidated.^122^ In terms of cholesterol efflux from the apical side, ABCG5 and ABCG8 have been found to play important roles, while other cholesterol transporters, such as ABCB1, may also be involved. Additionally, the secreted cholesterol may be reabsorbed at the apical side of enterocytes by NPC1L1.^123^

### 2.4 Cholesterol Oxidation

**Figure 4:**
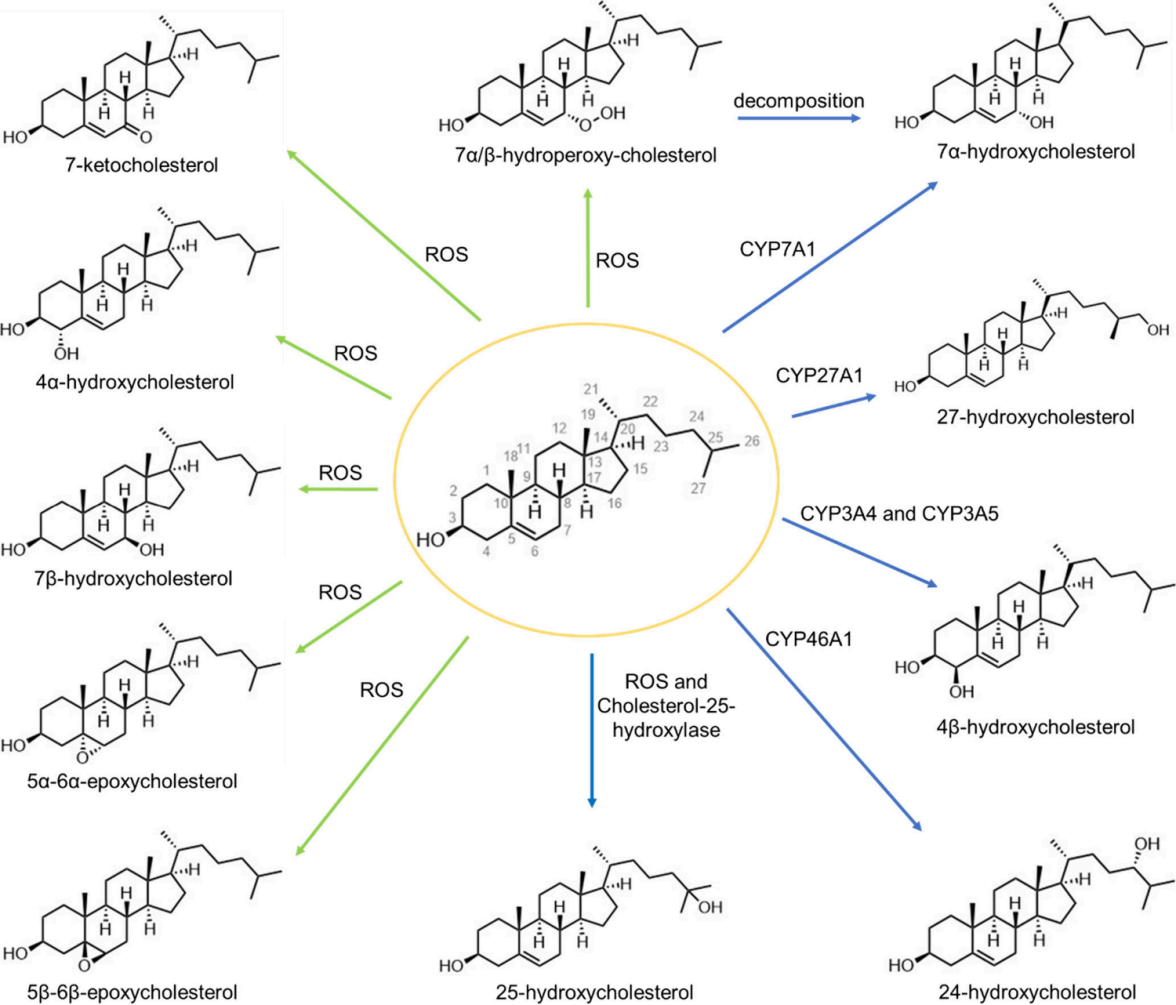
Major cholesterol metabolite production pathways.

Besides esterification, cholesterol is susceptible to multiple routes of oxidation. Over 70 cholesterol oxidation products have been identified that are either generated endogenously or are present in cholesterol-containing foods.^124^ Oxysterols are cholesterol oxidation products generated by auto-oxidation through exposure to oxygen, light, and heat, or by specific enzymatic oxidation.^24^ Cholesterol oxidation results in the addition of a hydroxyl, carbonyl, or epoxide functional group on the sterol nucleus or side chain of cholesterol (Figure 4).^125,126^ As oxidized derivatives of cholesterols, oxysterols act as intermediates of steroid hormones, 1,25-dihydroxyvitamin D3, and bile acids.^127^ Endogenous oxysterols are also potent regulators of cellular processes implicated in the pathogenesis of atherosclerosis, myocardial injury, Alzheimer’s disease, Parkinson’s disease, age-related macular degeneration, and many cancers.^126,128,129^ Reactive oxygen species (ROS), including peroxyl radicals generated by the leukocyte–H_2_O_2_–HOCl system during inflammation, can cause cholesterol oxidation into 7-ketocholesterol (7-KC), 7β-hydroxycholesterol (7β-HC), 4α-hydroxycholesterol, 5α-6α-epoxycholesterol, 5β-6β-peroxycholesterol, 7α/β-hydroperoxy-cholesterol, and 25-hydroxycholesterol.^125,127^ Cholesterol can also be oxidized enzymatically by cytochrome P450 enzymes, leading to the formation of 7α-HC by CYP7A1, 22-hydroxycholesterol (22-HC) by CYP11A1, 24-hydroxycholesterol (24-HC) by CYP46A1, 25-hydroxycholesterol (25-HC) by cholesterol-25-hydroxylase, 27-hydroxycholesterol (27-HC) by CYP27A1, and 4β-hydroxycholesterol (4β-HC) by CYP3A4 and CYP3A5.^125,127^ The 27-HC oxysterol has the highest concentration in circulation (67 to 199 ng/mL), and CYP27A1 is the most expressed enzyme in myeloid immune lineage cells.^125^ In macrophages, they regulate lipid transport and metabolism, cytotoxicity, and inflammatory responses.^127^ Macrophages cultured *in vitro* with cholesterol showed increased 27-HC secretion, while simvastatin, a cholesterol synthesis inhibitor, decreased 27-HC secretion by macrophages.^130^ This oxysterol can modulate immune responses by binding to Toll-like receptors (TLRs), such as TLR4 on macrophages,^129^ and induce production of IL-8,^131^ which has been linked to downstream activation of the CXCR2 pathway and tumor proliferation.^131,132^ Oxysterols in circulating oxidized LDL particles induce macrophages to become dysfunctional foam cells,^133,134^ and several oxysterols (27-HC, 7-KC, 7α-HC, and 7β-HC) have been found in foam cells.^135^

Importantly, up to 7.6 mg/mL of oxysterols have been found in commercial parenteral liposome formulations but not in the initial formulation materials, suggesting that they were generated during liposome manufacturing and/or storage.^136^ 7-KC (up to 0.95 ± 0.05 mg/mL), 7α-HC (up to 1.97 ± 0.86 mg/mL), 7β-HC (up to 1.36 ± 0.059 mg/mL), 5α,6α-epoxycholesterol (up to 0.12 ± 0.031 mg/mL), and 5β,6β-epoxycholesterol (up to 0.67 ± 0.07 mg/mL) were the major oxysterols found.^136^ Exposure to high temperature (e.g., 65 °C) and oxygen is typical during the manufacturing process and can potentially accelerate cholesterol oxidation.^136^ Strategies such as decreasing heat, lyophilization with reduced oxygen contact, and the addition of antioxidants also decreased cholesterol oxidation.^136^ 25-Hydroxycholesterol along with 5α,6α-epoxy-cholesterol and 5β,6β-epoxy-cholesterol have also been detected in liposomal formulations.^136,137^ Although the majority of oxysterols have been found to be highly bioactive (cytotoxic, immune modulatory, mutagenic, etc.); the biological levels that are considered safe have not been established. Moreover, the impact of these cholesterol oxidation products on the safety and efficacy of the LNP preparation has not been fully determined, although it is notable that 30–40 μmol/L (12–16 μg/mL) of 7β-hydroxycholesterol in patient serum has been reported as atherogenic.^138^

## 3 Immune Modulatory Oxysterols

Oxysterols have diverse biological effects, and many endogenous oxysterols are potent immune modulators. Increased inflammatory cytokines (IL-8, IL-1β, IL-6, IL-12, and TNFα) have been correlated with higher levels of oxysterols (7α-HC, 7β-HC, 7-KC, 27-HC, 25-HC, 5α,6α-epoxycholesterol, and 5β,6β-epoxycholesterol) in patients with atherosclerosis and in human monocytic cell lines, suggesting a link between oxysterols and enhancement of inflammatory processes.^139^ The immune modulatory effects of oxysterols are highly variable and involve different mechanisms. The major oxysterols and their impact on immune responses are summarized below. Notably, the vast majority of these studies were performed with isolated oxysterols, and it is likely that there will be differences in how LNP-derived oxysterols interact with cells and cellular components.

### 3.1 7-Ketocholesterol

7-Ketocholesterol (7-KC) is generated by non-enzymatic oxidation (e.g., in atherosclerotic lesions) and can be further internalized by macrophages.^140^ 7-KC is characterized by an additional ketone group at C7 which disrupts the packing organization of cell membrane lipids leading to increased membrane permeability.^140^ Increased levels of 7-KC are found in atherosclerotic plaques, and this oxysterol is responsible for an overproduction of ROS and cytokines, and is also associated with cell death in macrophages, endothelial cells, and smooth muscle cells.^141^ In fact, ROS can induce cell death through protein oxidation, lipid peroxidation, and mitochondrial and DNA damage mechanisms.^141−143^ Protein oxidation leads to reversible and irreversible oxidation of sulfhydryl (−SH) groups in proteins that are essential for the functionality of glutathione reductase, calcium ATPases, and actin, thus ultimately leading to cell death by apoptosis or autophagy.^144^

7-KC is the main oxidized cholesterol in oxLDL and has proinflammatory effects in macrophages through activation of multiple signaling pathways.^128^ Exposure of bone-marrow-derived macrophages (BMDMs) to 7-KC in the presence of LPS and interferon-γ (IFN-γ) stimulation increased proinflammatory markers compared to cholesterol exposure.^128^ Macrophage transcriptome analyses reveal that 7-KC regulates IL-17, TNF, and NF-κB signaling pathways and cytokine–cytokine receptor interactions.^128^ 7-KC is also an agonist of the liver X receptor (LXR), increasing the expression of target genes regulated by LXR that are related to lipid metabolism such as ABCA1, ABCG1, and SREBP-1c.^128^ However, the proinflammatory responses are mediated by mitochondrial ROS and the TLR4 mechanism, independent of LXR.^128^

### 3.2 24-Hydroxycholesterol

The blood–brain barrier prevents cholesterol transport, and *de novo* synthesis is utilized to produce cholesterol in the brain. The enzyme 24-hydroxylase (CYP46A1) is expressed by neural cells and produces 24-hydroxycholesterol (24-HC) from cholesterol,^145^ while excess cholesterol synthesized in the brain is transported out by its conversion to 24-HC. This oxysterol decreases RORγt, a transcription factor required for T helper cell differentiation into T helper 17 cells (Th17), and increases FOXP3 expression, a transcription factor for T regulatory cell differentiation.^146^ In the brain, 24-HC reduces the expression of IL-17A, granulocyte-macrophage colony-stimulating factor (GM-CSF), and macrophage inflammatory protein 3α (MIP-3α) and increases the expression of IL-10 and IFN-λ2.^146^ In the tumor microenvironment, hypoxia inducible factor-1a (HIF-1α) can increase expression of CYP46A1, thereby increasing 24-HC levels and inducing neutrophils to produce proangiogenic factors such as MMP9 and Bv8.^147^ In macrophages, 24-HC causes expression of proinflammatory molecules such as TNF by binding to integrin αvβ3 and activating the focal adhesion kinase and NFκB signaling.^148^

24-HC is a ligand for LXR and INSIG proteins and can further affect the immune system through these pathways.^149^ Besides being involved in macrophage cholesterol uptake/efflux and phagocytic activities, LXR also regulates T cell differentiation and autoimmune responses.^149^ INSIG proteins regulate the retention of sterol regulatory element binding proteins (SREBPs) in the endoplasmic reticulum (ER). SREBP is a transcription factor that regulates expression of genes involved in cholesterol and fatty acid synthesis.^149^ SREBP-1a has a high expression in immune cells and in macrophages. Dendritic cell activity is also regulated by LXR and SREBP effectors as LXR ligands can cause inhibition antigen presentation and T-cell priming/activation by dendritic cells, which can contribute to tumor cell induced immunosuppression.^149^

### 3.3 25-Hydroxycholesterol

The oxysterol 25-hydroxycholesterol (25-HC) is produced from cholesterol either through autoxidation or enzymatically through cholesterol-25-hydroxylase. Studies in murine macrophages (RAW 264.7 and BMDMs) and macrophages from healthy human volunteers show increased production of 25-HC in response to LPS,^150,151^ suggesting a role in the inflammatory response. 25-HC regulates cholesterol biosynthesis by binding and blocking the SREBP signaling, as well as by activating the nuclear receptor LXRα.^152^ Cholesterol-25-hydroxylase is activated by interferon (IFN) which induces the recruitment of signal transducer and activator of transcription 1 (STAT1) to the promoter region of the gene for cholesterol 25-hydroxylase.^153^ This interaction suggests a link between IFN stimulation, innate immune responses, and secretion of 25-HC by macrophages.^153^ Moreover, 25-HC regulates the type I IFN signaling pathway, decreasing IL-1β expression and inflammasome activation by antagonizing SREBP.^154^ Under hypoxia, 25-HC induces IL-8 production in human monocyte-derived macrophages, resulting in acceleration of atherosclerosis.^155^

### 3.4 7α-Hydroxycholesterol

7α-Hydroxycholesterol (7α-HC) is a major oxysterol derived from cholesterol through the rate limiting enzymatic action of CYP7A1 or 7α-hydroxylase.^156,157^ It can also be derived from cholesterol in a non-enzymatic manner through the action of ROS.^126^ 7α-HC modulates various aspects of the immune system, such as differentiation and migration of immune cells, as well as expression of inflammatory cytokines.^158,159^ In THP-1 monocytes, exposure to 7α-HC induced expression of mature dendritic cell (mDC) markers, including CD40, CD80, CD83, and CD88, suggesting a transitioning of monocytic cells into mature dendritic cells.^160^ 7α-HC can also induce the production of IL-8 (CXCL8) and chemokine receptor CCR5 ligands, CCL4 and CCL3, in macrophages.^159^ When incorporated into LNPs, 7α-HC enhances LNP uptake in T-cells, thereby presenting a potential application in immunotherapeutics.^161^

### 3.5 7β-Hydroxycholesterol

7β-Hydroxycholesterol (7β-HC), formed by cholesterol auto-oxidation, has cytotoxic and proapoptotic properties.^162^ 7β-HC induces the secretion of IL-1β and expression of adhesion molecules in vascular endothelial cells, similarly to 7-KC.^163^ In human NK cells, 7β-HC is associated with early lysosomal membrane permeabilization, ROS production, and late mitochondrial membrane permeabilization, resulting in apoptosis and necrosis.^164^ In human monocytes, 7β-HC induces the production of chemokines, such as MCP-1, MIP-1β, TNFα, IL-1β, and IL-8, which are associated with pro-inflammatory activity.^139^ The increase in IL-8 secretion appears to be due to an increase in intracellular calcium, which leads to activation of calcium-dependent kinases, such as ERK1/2, that subsequently activates AP-1, a transcription factor for IL-8.^165^ In macrophages, 7β-HC also increases oxidized levels of glutathione and decreases levels of heat shock protein 70 (HSP70), resulting in oxidative stress^166^ and macrophage apoptosis through ROS that is associated with accumulation of cytosolic lipid droplets and lysosomal destabilization.^167^ 7β-Hydroxycholesterol can also indirectly modulate immune responses through regulation of cortisol metabolism.^168^

### 3.6 27-Hydroxycholesterol

27-Hydroxycholesterol (27-HC) is one of the most abundant oxysterols in the blood and is generated by the CYP27A1 oxidation of cholesterol. This enzyme is expressed mainly in the liver and in myeloid immune lineage cells like macrophages.^169,170^ The expression of CYP27A1 in macrophages plays a role in cholesterol homeostasis and immunoregulation as 27-HC is a ligand of LXR.^171^ 27-HC enhances the expression of different genes including those involved in lipid metabolism, inflammation, and cell differentiation.^172^ 27-HC can influence the polarization state of monocytic cells via the LXR pathway by positive regulation of CD163 and CD206, markers of M2 functionality, while it negatively regulates the M1 markers CD80 and CD86 at the protein expression level.^172^ The shift toward M2-polarization induced by 27-HC is also associated with diminished CD36 and CD204 expression and increased expression of LXR, ABCA1, IL-10 (a cytokine associated with M2 functionality), and CCL2 (a chemokine that recruits monocytes and amplifies local inflammation).^160,173,174^ Additionally, 27-HC increases the transcription and secretion of CCR5 ligands, such as CCL3 and CCL4, in monocytic cells, which can promote the migration of CCR5-expressing Th1 cells.^175^

### 3.7 5,6-Epoxycholesterol

5,6-Epoxycholesterols (5,6-ECs) are generated in macrophages by free radical lipid peroxidation of cholesterol and result in a mixture of 5α,6α-epoxycholesteroland 5β,6β-epoxycholesterol diastereoisomers.^176−178^ Despite the presence of a three-member cyclic ether on the structure, which is highly electrophilic and known to have alkylating properties, the 5,6-ECs are stable and nonreactive toward nucleophilic macromolecules such as DNA.^179,180^ These epoxy-metabolites have immunomodulatory effects that were demonstrated in Wister rats fed a diet containing 5α,6α-EC, resulting in an increase in blood M1 macrophages and serum concentrations of cytokines, such as TNFα, IL-1β, and IL-6.^181^ Murine peritoneal macrophages show significantly higher accumulation of 5β,6β-EC under induced oxidative stress conditions, along with increased protein kinase C activity, superoxide anion release, and activation of the NADPH-oxidase system, thereby enhancing cell-mediated oxidation of LDL.^182^ 5,6-ECs can also induce apoptosis in human myeloid cell lines U937 and HL-60, through caspase-2L-mediated cell death.^183^

### 3.8 4-Hydroxycholesterol

While 4α-hydroxycholesterol (4α-HC) is generated by cholesterol autoxidation,^184^ 4β-hydroxycholesterol (4β-HC) is mainly produced by cytochrome P450 3A4 (CYP3A4)^185^ and CYP3A5.^184^ Induction of CYP3A4 by drugs such as antiepileptics,^184,185^ as well as by activation of pregnane X receptor (PXR), increases 4β-HC production.^185^ 4β-HC, but not its isomer 4α-HC, has been reported to activate LXRα and LXRβ.^185,186^ In human primary monocyte-derived macrophages and foam cells, 4β-HC represses cholesterol influx by LOX-1 and decreases expression of IDOL, a major regulator of LDLR, but it had no effects on CD36 and SR-AI.^110^ Concomitantly, 4β-HC increases cholesterol efflux by inducing the expression of ABCA1 and ABCG1.^110^ 4β-HC also stimulates triglyceride synthesis and accumulation of lipid droplets by inducing SREBP1c, a major transcription factor regulator of fatty acid and triglyceride synthesis, and also an agonist of LXR.^103^ Considering its lack of activation of SREBP2, 4β-HC does not trigger cholesterol synthesis; instead, it is responsible for shifting the lipid homeostasis to triglyceride accumulation.^103^ ROS production associated with 4β-HC is similar in order of magnitude as 25-HC, but 4β-HC does not appear to induce inflammatory cytokines as 25-HC does.^186^

### 3.9 Secondary Oxysterols

7α,25-Dihydroxycholesterol (7α,25-diHC) is linked to multiple sclerosis and chronic obstructive pulmonary disease (COPD) but not atherosclerosis.^127^ It is a secondary oxysterol formed by CYP7B1 hydroxylation of 25-HC or through CYP7A1 hydroxylation of cholesterol into 7α-hydroxylation with subsequent 25-hydroxylation.^127^ This metabolite is a ligand for the Epstein–Barr virus-induced gene 2 (EBI2), C-X-C Motif Chemokine Receptor 2 (CXCR2), G-protein-coupled receptor 17 (GPR17), and the smoothened receptor.^127^ The enzymes involved in the formation of 7α,25-diHC are highly regulated during inflammatory processes.^127^ While 25-HC is almost an inactive ligand of EBI2, 7α,25-diHC is considered to be its most active ligand. In response to 7α,25-diHC exposure, human monocyte-derived macrophages show an upregulation of EBI2, cholesterol 25-hydroxylase, CYP7B1, and CYP7A1 expression and an increase in migration and production and release of oxysterols into the cellular environment.^187,188^

7β,27-Dihydroxycholesterol (7β,27-diHC), another secondary oxysterol, is generated through CYP27A1 hydroxylation of 7β-hydroxycholesterol.^189^ A similar secondary oxysterol, 7α,27-dihydroxycholesterol (7α,27-diHC), is produced by CYP7B1 oxidation of 27-HC.^190^ Both 7β,27-diHC and 7α,27-diHC are potent RORγt agonist ligands.^190^ They function as endogenous drivers of CD4^+^ Th17 T cell differentiation. T cells activated under Th17 conditions were associated with increased production of 7β,27-diHC and 7α,27-diHC and subsequent Th17 differentiation and IL-17 production.^191^ It is possible that a balance between high level oxysterols, such as 27-HC, 7α-HC, and 24(S)-HC, and low-level oxysterols, such as 7β,27-diHC and 7α,27-diHC, needs to be maintained at a steady level in order to keep homeostasis of IL-17 producing cells (CD4^+^ Th17 cells and γδ^+^ T cells).^191^ In CYP27A1 knockout mice, the disruption in the production of 7α,27-diHC causes impairment of dendritic cell migration and maintenance.^192−194^ 7α,27-diHC and 7α,25-diHC are both also ligands for EBI2; however, the *in vitro* ligand activity of 7α,27-diHC is 10 times lower than that of 7α,25-diHC.

## 4 Mechanisms of Immune Modulation by Oxysterols

### 4.1 Liver X Receptor (LXR)

**Figure 5:**
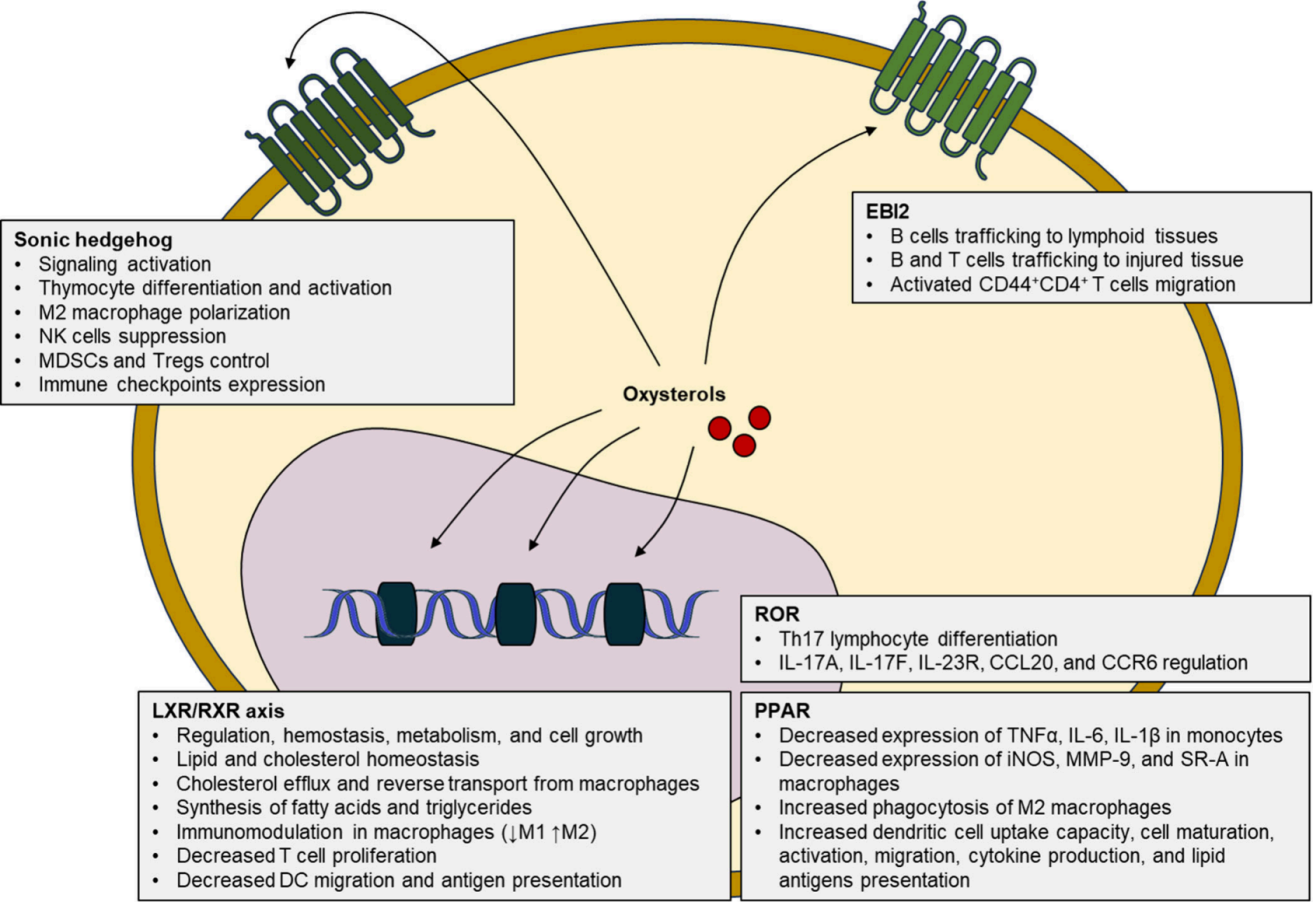
Primary pathways linking lipid metabolism and immune responses promoted by oxysterols. PPAR, peroxisome proliferator-activated receptors; LXR, liver X receptor; RXR, retinoid X receptor; ROR, retinoic acid receptor-related orphan receptor; EBI2, Epstein–Barr virus induced gene 2; NK cells, natural killer cells; MDSCs, myeloid-derived suppressor cells; Tregs, regulatory T cells; DC, dendritic cells.

There are several cellular pathways that cross-talk between lipid metabolism and inflammatory signaling through interactions with oxysterols (Figure 5). Among these, the liver X receptor (LXR) is the most well-studied.^126,195^ The two isoforms of this receptor, LXRα (NR1H3) and LXRβ (NR1H2), are nuclear receptors belonging to a superfamily of 48 ligand dependent transcription factors. LXRβ is widely expressed across all tissues, but LXRα is tissue specific, being mostly expressed in the liver, adipose tissue, adrenal glands, intestine, lungs, and cells of myelomonocytic lineage.^196^ They are involved in developmental regulation, hemostasis, metabolism, and cell growth. For instance, LXRs are responsible for increasing cholesterol efflux and reverse cholesterol transport from macrophages, thus being involved in lipid and cholesterol hemostasis. By dimerization with the retinoid X receptor (RXR),^61,103^ these receptors enhance the product of certain genes, such as ABCA1, ABCG1, and ApoE, which regulate the efflux of cholesterol, as well as SREBP-1c, lipoprotein lipase (LPL), and fatty acid synthase (FAS), which increase fatty acid uptake, synthesis, and triglyceride production.^197^ This dimerization also facilitates the excretion of cholesterol from the body by activating genes, such as cytochrome p450 7α-hydroxylase, to convert cholesterol into bile acids.^103^

Oxysterols are endogenous ligands of LXR;^68^ however, different oxysterols have different effects on LXR activity. The presence of a hydroxyl at carbon 4, 7, 20, 22, 24, 26, 25, or 27 in the molecule renders LXRα activity, while a hydroxyl at carbon 5, 6, 12, 16, or 17 leads to LXRα inactivation.^152^ Notably, the oxysterol 7a,25-OHC does not activate LXRs as compared to the other structurally related oxysterols, such as 22(R)-HC, 24-HC, and 25-HC.^86^

Oxysterol activation of LXR has an overall inhibitory effect on immune responses. In macrophages, it promotes survival and downregulation of M1-associated genes^127,198^ such as IL-6, IL-1β, MCP-1, MCP-3, iNOS, and COX-2,^127,199^ while upregulating M2-associated genes such as Arg1 and IL-10.^173,200^ Additionally, LXR activation negatively affects activated T and B cells.^198^ In T cells, it leads to inhibition of IL-2 and IL-7 induced T cell proliferation,^201^ while, in differentiating B cells, it decreases IgE secretion.^202^ LXRs are also involved in the differentiation of Th17 cells since abnormal gene expression of LXR prevented Th17 polarization in murine CD4^+^ T cells and LXR deficiency induced Th17 differentiation *in vitro*.^203^ Moreover, the expression of fascin, a protein responsible for bundling actin fibers, is disrupted by the activation of LXRα during the differentiation process of monocyte derived DCs, which consequently results in a reduction in their T-cell activation capacity.^204^ Additionally, during DC maturation, the activation of LXRα impedes chemokine receptor CCR7 expression, resulting in inhibition of DC migration toward the chemokine CCL19, while silencing of LXRα partially reverses the downregulation of CCR7 by oxysterols.^144^

### 4.2 Retinoic Acid Receptor-Related Orphan Receptor (ROR)

The retinoic acid receptor-related orphan receptors (RORs) are part of the nuclear receptor family of transcription factors which oxysterols can bind.^205^ They are composed of three different main subtypes: RORα, RORβ, and RORγ, also referred to as NR1F1, NR1F2, and NR1F3, respectively.^205^ Activation of gene transcription occurs when the RORs bind to the specific ROR response elements on DNA and recruit coactivators.^205^ Unlike RORβ for which there is no oxysterol found so far to bind to it, there are several other oxysterols such as 25-HC, (25R)-26-HC, and 7α-HC that bind to RORα and RORγ.^205^ (25R)-26-HC is a secondary oxysterol formed from the primary oxysterol (25R)-26-HC, and it is characterized by a hydroxy group at the 26th carbon atom with a specific chiral configuration (R) at the 25th carbon atom.^206^ The RORα gene encodes four human isoforms which have a conserved DNA binding domain, ligand binding domain, hinge domain, and different distinct N-terminal domains.^205^ RORα is expressed in the liver, skin, lung, brain, adipose tissue, and muscle, regulating lipid and glucose metabolism and circadian rhythm.^205,206^ A gene known as *Rorg* encodes both RORγt and its isoform RORγ. Although their mRNA differs in the first 100 nucleotides, they have the same DNA and ligand binding domains.^207^ The first isoform is widely expressed across various organs, including the kidney, liver, and muscles. It plays a crucial role in facilitating the differentiation of T cells into Th17 and is essential for the transcriptional regulation of the IL-17A gene.^208^ In contrast, RORγt is expressed preferably in the thymus and is also involved in the differentiation of Th17 cells. The essential genes associated with Th17 cell functionality, such as IL-17A, IL-17F, IL-23R, CCL20, and CCR6, are regulated by both RORγt and RORα.^207^ There are several endogenous oxysterols, such as 7β,27-diHC and 7α,27-diHC, that have been identified as RORγt agonists. Among them, 7β,27-diHC is the most selective and potent RORγt activator. Th17 cells can produce these two oxysterols, and the administration of 7β,27-diHC to mice also results in excess production of IL-17.^191^ Concomitantly, in mice with CYP27A1 deficiency or RORγt deficiency, there is a decrease in the number of IL-17 producing cells, such as CD4^+^ and γδ^+^ T cells.^191^ Overall, this suggests a connection between the production of oxysterols and the generation of IL-17-producing immune cells.

### 4.3 Peroxisome Proliferator-Activated Receptors (PPARs)

Peroxisome proliferator-activated receptors (PPARs) make up a group of nuclear receptors that enhance ligand-dependent transcription of target genes. They comprise three subtypes: PPARα, PPARγ, and PPARβ/δ. This receptor family regulates cellular metabolic function and energy balance.^209,210^ PPARα is preferentially expressed in tissues associated with fatty acid oxidation such as liver, kidney, heart, brown fat, and skeletal muscle. While the expression of PPARδ is ubiquitous, PPARγ is predominantly expressed in adipose tissue, colon, and monocytes/macrophages.^211^ PPARγ activation triggers M2 macrophages to increase phagocytosis of apoptotic cells.^212^ PPARγ is also highly expressed in dendritic cells (DCs), affecting their antigen uptake capacity, cell maturation, activation, migration, cytokine production, and lipid antigen presentation.^213^ Additionally, PPARγ ligands inhibit the expression of TNFα, IL-6, and IL-1β in monocytes, as well as inducible nitric oxide synthase (iNOS), matrix metalloprotease-9 (MMP-9), and scavenger receptor-A (SR-A) expression in macrophages.^214^ The oxysterol 25-hydroxycholesterol-3-sulfate (25HC3S) is a PPARγ ligand, suggesting a mechanism that links lipid metabolism to immune responses. In macrophages, this oxysterol activates the PPARγ/IκB/NF-κB signaling pathway and results in suppression of inflammatory responses.^215^ Similarly, in endothelial cells, PPARγ activators show an inhibitory effect on the expression of the CXC chemokine IFN-inducible protein of 10 kDa (IP-10), monokine induced by IFN-γ (Mig), and IFN-inducible T-cell α-chemoattractant (I-TAC) in response to IFN-γ stimulation.^216^ This occurs through a decrease in the promoter activity of IP-10 and through the inhibition of protein binding to NF-κB.^216^ Thus, the overall effect of oxysterols is to suppress inflammatory immune responses through PPAR pathways.

### 4.4 Sonic Hedgehog Signaling

Cholesterol and its metabolites are important modulators of the Sonic hedgehog (Shh) signaling pathway. The Shh signaling pathway regulates tissue development during embryogenesis and plays critical roles in regenerative responses to injury.^217,218^ The Shh protein is covalently modified by cholesterol at the N-terminal fragment.^219^ This modification is required for Shh to be fully active. Genetic defects in the sterol synthetic pathway lead to holoprosencephaly,^220^ which has been associated with Shh deficiency.^221^ One early inhibitor of the Shh pathway, cyclopamine, is a sterol-like plant alkaloid that binds the smoothened receptor (Smo) within the seven-transmembrane domain.^222^ Oxysterols with hydroxyl groups on the isooctyl side chain of cholesterol also activate Shh signaling by binding to the extracellular cysteine-rich domain of Smo,^223,224^ with 20(S)-hydroxycholesterol as the most potent oxysterol ligand.^225,226^ The Shh signal is transduced across the cell membrane by Smo, which is inhibited by another transmembrane protein, called Patched (Ptch). Ptch functions both as the Shh receptor and as a tumor suppressor: the binding of a Shh protein ligand inhibits Ptch, resulting in the activation of Smo and the transcription of Shh target genes driven by the Gli transcription factors. Aberrant activation of the hedgehog signaling pathway has been linked to the development of various types of human cancers, such as basal cell carcinoma, medulloblastoma, and breast cancer.^227^ Increasing evidence also suggests that Shh signaling is involved in tumor immune evasion and poor responses to cancer immunotherapy by suppressing the immune system and promoting an immunosuppressive tumor microenvironment. For example, Shh signaling is important during thymocyte differentiation and activation.^228^ It also plays an important role in promoting M2 polarization of TAMs,^229^ suppressing natural killer cells,^230^ and controlling myeloid-derived suppressor cells (MDSCs)^231^ and regulatory T cells (Tregs).^232,233^ Thus, there is a potential for oxysterols that activate Shh to suppress antitumor immunity and promote tumor progression, whereas strategies to inhibit Shh signaling have anticancer effects. Vismodegib and sonidegib are two Smo inhibitors approved by the FDA to treat basal cell carcinoma, while glasdegib is approved for acute myeloid leukemia in combination with low-dose cytarabine.^234^ In addition, Shh signaling promotes immunosuppression by inducing the expression of immune checkpoint molecules including PD-1/PD-L1,^235−237^ and the combinatorial use of Shh pathway inhibitors and immune checkpoint inhibitors as anticancer therapeutics has been actively investigated in multiple clinical trials^238^ (https://clinicaltrials.gov; NCT03521830, NCT05538091, and NCT02690948).

### 4.5 Epstein–Barr Virus Induced Gene 2 (EBI2)

The Epstein–Barr virus induced gene 2 (EBI2) is a G-protein-coupled receptor that is mainly expressed in B and T cells.^239^ This gene regulates the trafficking of B cells in lymphoid tissues and is essential for producing humoral immune responses.^239,240^ EBI2 is activated by oxysterols, among which 7α,25-diHC is the most potent ligand.^193,194,239^ Oxysterol binding to EBI2 results in activation of Gαi (subunit of G protein), recruitment of β-arrestin, and migration of EBI2-expressing B and T cells to specific locations.^239,241^ In mice that are EBI2 deficient, there is reduced B cell migration to the outer follicle of secondary lymphoid tissues and diminished antibody responses to T-dependent antigens.^239,241^ The overexpression of EBI2 is sufficient to induce localization of B cells to the outer follicle.^240^ Oxysterols, particularly 7α,25-diHC, can also enhance the migration of activated CD44^+^CD4^+^ T cells by interacting with EBI2 in inflamed tissues;^242^ therefore, EBI2 is considered a chemoattractant receptor.^157^

## 5 Implications and Future Research Directions for Cholesterol-Containing LNPs

There is increasing evidence that oxysterols play a critical role in the proliferation of malignant cells. Cancer cells require large amounts of cholesterol for membrane synthesis; consequently, LDLRs are upregulated to increase the uptake of cholesterol-rich LDL particles.^24^ Concomitantly, the expression of LXRs is reduced in tumor cells, with LXR activation associated with decreased proliferation of colorectal, gallbladder, prostate, breast, and ovarian cancer, as well as glioblastoma, melanoma, and leukemia.^126^ Additionally, the tumor microenvironment is infiltrated by immune cells, with tumor-associated macrophages (TAMs) as the predominant immune cells mediating immune evasion and inflammation.^76^ LNP-encapsulated doxorubicin accumulated in TAMs to a greater extent than free doxorubicin, and this was reported to be also higher in M2 than M1 polarized TAM,^243^ indicating that LNPs passively target TAMs and that macrophage functionality plays a role. It is possible that LNP uptake polarized macrophages toward an M2-phenotype. Similar cholesterol-containing LNPs have been found to promote TAM infiltration and tumor angiogenesis, inhibit T cell responses, and increase tumor growth in some murine tumor models.^244,245^ Although the precise molecular mechanisms are unknown, it is possible that LNPs accumulating in tumor tissues generate oxysterols in the cellular environment. Tumor-derived oxysterols are capable of recruiting protumoral macrophages and neutrophils, thereby inhibiting antitumor immune responses.^126^ Macrophages also secrete oxysterols, which can then be taken up by other immune cells such as lymphocytes, inhibiting their proliferation. Increased lipids in the environment of tumor cells can lead to their incorporation into cell membranes and tumor cell proliferation.^246−248^ Notably, the vast majority of the studies in this field were performed with isolated oxysterols, and it is possible that there will be differences in how LNPs interact with cells and cellular components such as membranes, enzymes, and receptors compared to endogenous oxysterols. Nonetheless, the role of exogenous LNP-associated oxysterols in the pathophysiology of cancer and cancer-associated immunosuppression has yet to be precisely established.^195^

The pivotal role of oxysterol in immunoregulation and cancer also implies the therapeutic potential of oxysterol targeting LNPs, such as sHDL nanoparticles. Similar to native HDL, sHDL nanoparticles could effectively induce cholesterol and oxysterol efflux from the cells. Moreover, the antioxidative effects of sHDL nanoparticles may be leveraged to inhibit the production of oxysterol.^249^ The antioxysterol effects of sHDLs have been proved by earlier studies where plasma isolated HDL or sHDL nanoparticles significantly reduced 7-KC induced cellular dysfunction on endothelial cells and macrophages.^250,251^ Additionally, sHDL could be further enhanced by encapsulating LXR agonists in the nanoparticles, which resulted in higher cholesterol and oxysterol efflux capacity, as well as more potent anti-inflammatory effects.^252^ Nevertheless, more studies are needed to maximize the immunoregulatory effects of oxysterol scavenging LNPs in broader disease areas, such as cancer and inflammatory and cardiovascular diseases.^27^

The natural cholesterol transport pathways have inspired strategies to optimize the *in vivo* pharmacokinetics and efficacy of LNPs. For instance, modifying nanoparticles with ApoE derived peptides could significantly increase the LNP uptake by macrophages, improving the drug delivery efficiency to atherosclerotic plaques.^253^ ApoE functionalization also enhanced transport across the blood–brain barrier, which may present a promising strategy for brain drug delivery.^254^ Apolipoprotein can also be introduced to LNPs through the protein corona. For example, one study reported that local adsorption of ApoB48 in intestinal tissue could promote the nanoparticle uptake by macrophages, leading to the improved anticancer effects through the tumor homing ability of macrophages.^255^ Further investigations on the interplays among LNPs, apolipoproteins, and macrophages would be valuable to informing the rational design of LNPs.

Similarly, replacing LNP cholesterol by cholesterol analogues that do not undergo metabolization into oxysterols can be a potential alternative to mitigate undesirable immune response. For instance, liposomes containing β-sitosterol, a naturally occurring cholesterol in plants, have shown negligible structural differences in the lipid bilayer compared to those containing cholesterol.^256^ More importantly, β-sitosterol reduces cholesterol metabolism and inflammatory responses, increases endogenous antioxidant defense,^257,258^ and has anticancer activity.^259^ Interestingly, in LNPs, cholesterol analogues with an alkyl substitution at C24, such as β-sitosterol, promoted structural modification and cellular uptake and retention and increased gene delivery, suggesting different lipid trafficking.^27^ Other potential analogues include epicholesterol, lanthosterol, cholestenone, cholestanol, stigmasterol, fucosterol, campesterol, β-sitostanol, stigmastanol, and ergosterol. Structure–activity studies to better understand the immune modulatory effects and metabolic pathways of cholesterol and its analogues will be critical for the design of synthetic cholesterol analogues with tunable immune modulation,

The preclinical evaluation of the immunological effects of liposomes has historically relied on *in vitro* studies and short-term studies in animal models, which are best suited for evaluating acute toxicities, such as those attributed to blood complement activation and production of cytokines. In contrast, immunosuppressive effects, especially those that affect the adaptive immune system, tend to manifest after longer periods and require more complex *in vivo* immunological assessments, which have not been widely incorporated into the preclinical drug development paradigm. The dearth of *in vivo* and long-term immunological studies during preclinical drug development presents a major barrier to fully realize the clinical potential of LNPs.^260^ Long-term immunosuppression associated with other therapeutics, such as corticosteroids, has been shown to have a detrimental impact on overall health and therapeutic outcomes. As LNPs are increasingly being investigated for applications in chronic conditions such as atherosclerosis, there is an imperative need to better understand the long-term consequences of LNP-associated immune modulation.

## 6 Conclusion

Cholesterol homeostasis and metabolism are pivotal in immunoregulation. In addition to readily engulfing LNPs,^261,262^ the high expression of cholesterol hydroxylases and ROS in macrophages^263^ suggests that they will readily produce oxysterols from LNP-associated cholesterol. Yet, the *in vivo* metabolic fate of LNP-associated cholesterol remains unclear due to a paucity of systematic studies. The prevailing paradigm in lipid biology positions oxysterols as highly potent immunoregulators, suggesting that it would be imperative for the drug delivery field to better understand the *in vivo* fate of LNP-associated cholesterol and the underlying mechanisms that are common to both immune responses and cholesterol metabolism.

## Vocabulary

Lipid nanoparticles: nanometer-scale structures typically composed of phospholipids and cholesterol.
Macrophage: a phagocytic white blood cell responsible for eliminating foreign substances, dead cells, and pathogens by engulfment, and also involved in antigen-presentation and activation of other immune cells.
M1 macrophage: macrophages that are activated toward a functional state that is characterized by secretion of pro-inflammatory cytokines and chemokines, capable of activating other immune cell types; also called classically activated macrophages.
M2 macrophage: macrophages that are activated toward a functional state that is involved in anti-inflammatory, pro-healing, and pro-angiogenic responses; also called alternatively activated macrophages.
Foam cells: a type of macrophage characterized by accumulation of lipid droplets in the cytoplasm commonly present in diseases such as atherosclerosis.
Oxysterols: products generated from cholesterol enzymatic oxidation or auto-oxidation.
